# Risk Stratification in HPV-Associated Oropharyngeal Cancer: Limitations of Current Approaches and the Search for Better Solutions

**DOI:** 10.3390/cancers17030357

**Published:** 2025-01-22

**Authors:** Bailey Fabiny Garb, Elham Mohebbi, Maria Lawas, Shaomiao Xia, Garett Maag, Peter H. Ahn, Nisha J. D’Silva, Laura S. Rozek, Maureen A. Sartor

**Affiliations:** 1 Department of Computational Medicine and Bioinformatics, University of Michigan, Ann Arbor, MI 48109, USA; bgarb@umich.edu (B.F.G.);; 2 Lombardi Comprehensive Cancer Center, Georgetown University, Washington, DC 20057, USAlr898@georgetown.edu (L.S.R.); 3 Department of Radiation Oncology, MedStar Georgetown University Hospital, Washington, DC 20007, USA; 4 Department of Periodontics and Oral Medicine, University of Michigan, Ann Arbor, MI 48019, USA; njdsilva@umich.edu; 5 Rogel Cancer Center, University of Michigan, Ann Arbor, MI 48109, USA; 6 Biostatistics Department, University of Michigan, Ann Arbor, MI 48109, USA

**Keywords:** human papillomavirus, oropharyngeal cancer, de-intensification, precision oncology, molecular biomarkers

## Abstract

The rising incidence of human papillomavirus (HPV)-associated oropharyngeal squamous cell carcinoma (OPSCC) necessitates advancements in risk stratification to optimize treatment outcomes and improve the quality of life for patients. Despite its favorable prognosis compared to HPV-negative OPSCC, current clinical staging and biomarkers, such as p16 status, are limited in their ability to distinguish between high- and low-risk patients within HPV-associated OPSCC. This limitation results in the overtreatment of low-risk patients, exposing them to unnecessary toxicity, and the undertreatment of high-risk patients who require more aggressive interventions. This review critically evaluates current stratification methods, including clinical assessments, de-escalation trials, and candidate molecular biomarkers for risk stratification. Emerging approaches such as immune markers, viral genomic integration patterns, and other molecular markers offer promising avenues for enhanced prognostic accuracy. By integrating advanced risk stratification methods, tailored treatment approaches may one day be developed to balance oncologic efficacy with reduced treatment-related morbidity. This review underscores the need for continued research into predictive biomarkers and adaptive treatment strategies to better address the diverse risk profiles of HPV-associated OPSCC patients.

## Simple Summary

Oropharyngeal cancers are increasingly caused by infections with high-risk types of human papillomavirus (HPV). Although the survival rates are high for this type of cancer, survivors suffer from treatment-related toxicities and long-term reduced quality of life. Some progress has been made towards precision treatment based on individual risk profiles; however, there is still room for improvement in this area, as molecular markers are not yet utilized. Here, we review current stratification approaches for HPV-associated oropharyngeal cancers, including recent de-escalation trials and candidate molecular biomarkers for risk stratification.

## 1. Introduction

The epidemiology of oropharyngeal squamous cell carcinoma (OPSCC) is evolving, with consequences for the treatment and prevention of this disease. OPSCC is a subset of head and neck squamous cell carcinomas (HNSCCs) that typically occur in the tonsils, at the base of the tongue, soft palate, and posterior and lateral pharyngeal walls. Over the past three decades, the primary cause of OPSCC has shifted from carcinogens like tobacco and alcohol to human papillomavirus (HPV) infection [1,2]. This trend has been observed globally including in the US, the UK, Denmark, Germany, Sweden, Japan, South Korean, and China [3,4,5,6,7,8,9,10]. HPV-associated OPSCC presents unique molecular and genetic profiles, immune infiltration, and responses to treatment compared to HPV-negative OPSCC [11,12,13]. The two main HPV oncogenes, E6 and E7, drive carcinogenesis through the degradation of the tumor suppressors p53 and Rb, respectively, leading to unchecked cell cycle progression and genomic instability.

The eighth AJCC manual on TMN staging addressed the biologic differences in HPV-driven OPSCC, including staging based on HPV status. Recognizing the overexpression of p16 in the course of the disease, AJCC recommended p16 immunohistochemistry (IHC), as the p16 status is commonly employed as a surrogate biomarker for HPV-associated OPSCC due to its strong sensitivity (approximately 94%) in identifying HPV-positive tumors [14]. However, p16 IHC specificity is lower, and depending on the attributable fraction of HPV in the population, its positive predictive value is highly variable, leading to the critical misclassification of a minority of patients who are p16-positive but HPV-negative (p16 IHC+/HPV−) [15]. The discrepancy may arise for a few reasons—false-positive p16 IHC or the overexpression of the p16INK4a tumor suppressor protein—which can occur in contexts other than HPV-driven malignancies [16]. p16 IHC+/HPV− patients risk being under-treated, especially in de-escalation trials designed to reduce the intensity of treatment for HPV-positive tumors. Thus, while p16 is a valuable tool for initial stratification, AJCC recognizes further refinement, with HPV DNA or RNA testing being recommended to avoid compromising patient outcomes in clinical settings [17,18].

While HPV-associated OPSCC accounts for approximately 26% of incident OPSCCs globally, the attributable fraction differs greatly across regions, with figures ranging from 5% in Chile to 85% in Finland and Sweden [19,20,21,22]. In both the UK and the USA, the rate of oropharyngeal cancer among men has exceeded that of cervical cancer in women [4]. Current estimates suggest that HPV is responsible for approximately 71% of OPSCC cases in the USA and 52% in the UK, with HPV-16 being the most common high-risk HPV strain identified among OPSCC patients [23,24]. According to the most recent International Agency for Research on Cancer (IARC) report, by 2040, the global incidence and mortality rates of oropharyngeal cancer are anticipated to rise by approximately 40% [25]. This increase is most pronounced among men, who are more likely to develop the disease compared to women [26]. A variety of risk factors exist for HPV-associated OPSCC, including oral sexual activity, tobacco and alcohol use, age, and oral hygiene [27,28]. In addition, co-infection with other viruses like Epstein–Barr, herpes simplex virus, and human immunodeficiency virus have effects on replication, persistence, and progression in HPV-related disease [29].

Patients with HPV-associated OPSCC have higher 5-year survival rates than those with HPV-negative OPSCC. Although estimates vary, the survival advantage of HPV is approximately 28% [30,31]. Despite the increased survival compared to HPV-negative patients, HPV-associated OPSCC patients have high levels of long-term toxicity and poor quality of life (QoL) [32,33]. Standard treatments, which involve surgery, high doses of radiation, and chemotherapy, can result in significant adverse effects. Common complications include dysphagia, xerostomia, and overall long-term reduction in QoL. Traditional high-intensity treatment often leads to persistent issues with speech, swallowing, and social functioning, especially for younger patients expected to live for several decades post-treatment [34,35]. Studies, such as those exploring de-intensification regimens like neoadjuvant chemotherapy with surgery, report that patients receiving standard therapies often experience appearance-related distress and social challenges despite near-normal recovery in other areas of function [34,36]. These findings underscore the importance of personalized de-intensification to improve long-term QoL while maintaining survival. Thus, while current treatment regimens provide excellent survival rates, they do not adequately account for differences in risk within the HPV-associated population. Many low-risk HPV-associated patients may be over-treated, leading to unnecessary side effects, while high-risk patients may still need aggressive interventions. More robust risk stratification can help tailor treatment intensity appropriately, leading to better survival and higher QoL.

Here, we discuss the current methods to stratify HPV-associated patients into high- and low-risk groups, including methods recently used in de-intensification trials and novel biomarkers that are candidates for future clinical trials. Our goal is to inform researchers who focus on biomarker discovery or translational research for risk stratification of HPV-associated OPSCC, so that novel biomarkers are developed taking into account the current framework of risk stratification. We begin by reviewing adjuvant, neoadjuvant, and definitive clinical trials, the earliest of which led the way for optimizing treatment protocols specific for HPV-associated OPSCC patients. We review trials that used various inclusion criteria to stratify patients for reduced postoperative radiation doses, preoperative treatments, and various chemotherapy regimens. For example, the superiority of the platinum-based chemotherapy drug cisplatin over the anti-EGFR antibody targeted cancer drug, cetuximab, was shown for HPV-associated OPSCC. Follow-up trials, some of which failed to achieve their objective, began fine-tuning treatment strategies while demonstrating the importance of patient selection. The most recent trials are beginning to incorporate imaging or molecular biomarkers, either as inclusion criteria or to guide adaptive treatment strategies. Finally, we review a variety of cellular and molecular biomarkers that are potential candidates for further risk stratification in clinical trials. For example, several markers for immune infiltration have shown the ability to stratify HPV+ OPSCC patients prior to treatment.

## 2. De-Intensification Trials

In HPV-associated OPSCC, advancements in treatment strategies have increasingly focused on balancing effective oncologic control with minimizing treatment-related toxicities. This has led to the exploration of de-intensified and adaptive treatment approaches, including adjuvant clinical trials, neoadjuvant strategies, definitive chemoradiation trials, and biomarker-driven trials. These approaches aim to address the unique responsiveness of HPV-associated OPSCC patients to therapy while tailoring treatments to individual risk profiles. This section reviews the findings and implications of major clinical trials and emerging approaches in HPV-associated OPSCC, shedding light on the challenges and future directions for de-intensification and adaptive treatment strategies. By incorporating risk stratification, novel biomarkers, and advanced therapeutic modalities, these strategies aim to redefine the standard of care for this unique patient population, optimizing outcomes while prioritizing quality of life.

### 2.1. Adjuvant Clinical Trials

Adjuvant clinical trials in HPV-associated OPSCC have explored risk-adapted approaches to optimize postoperative therapy, aiming to balance oncologic efficacy with reduced treatment toxicity. In the DIREKHT trial [37] (2014–2021), 150 patients with a mix of HPV-associated and HPV-negative HNSCCs were stratified post-surgery into three groups based on factors including resection margins, TNM stages, tumor stages, and peritumoral lymphangiosis (Table 1). Patients at low risk for local recurrence received reduced radiation doses (from 60–66 Gy to 56 Gy), those at low risk for contralateral neck node metastases omitted contralateral neck irradiation, and those at low risk for both local recurrence and contralateral neck node metastases received both the dose reduction in the primary tumor region to 56 Gy and the omission of elective contralateral neck radiotherapy. The results showed the cumulative local reginal recurrence (LRR) was low at 6% after 1, 2, and 3 years, with oropharyngeal cancer patients showing particularly favorable outcomes (1% LRR), while oral cavity cancer patients exhibited higher recurrence rates (14% LRR), highlighting the need for cautious patient selection. DFS rates for all patients, regardless of their HPV status, were 95%, 92%, and 89% at 1, 2, and 3 years, and OS remained high at 96%, 94%, and 94% over the same period. De-intensified therapy resulted in moderate-to-severe acute toxicities; however, these toxicities significantly improved over time: grade 3 dysphagia decreased from 11% at 9 months to 1% at 27 months, and grade 3 xerostomia resolved entirely (2% to 0%). While concurrent chemotherapy elevated acute toxicity rates, late toxicities remained comparable. These findings indicate that de-intensified therapy is associated with substantial but recoverable acute toxicity, with excellent long-term outcomes improving the QoL for most patients. These results support the use of de-intensified regimens in appropriately stratified patients to balance excellent oncologic control with a reduced treatment burden, although higher recurrence rates in oral cavity cancers underscored the importance of careful risk assessment. The DIREKHT trial’s inclusion of patients irrespective of HPV status distinguished it from the de-intensification studies that focused exclusively on HPV-associated OPSCC. By broadening the scope, the trial aimed to explore the feasibility of de-intensified radiotherapy across a more diverse patient population. However, the lack of stratified reporting based on HPV status limits the ability to discern specific outcomes for this biologically distinct group.

The ECOG-ACRIN E3311 trial [38] (2013–2017) investigated risk-stratified postoperative radiation therapy (PORT) in patients with resected stage III-IVa (AJCC-v7) HPV-associated OPSCC. Patients were categorized into low-risk, intermediate-risk, and high-risk groups based on tumor and nodal pathologies, surgical margins, lymphovascular invasion, perineural invasion, and extranodal extension (ENE) (Table 1). Low-risk patients underwent observation only, intermediate-risk patients were randomized to receive 50 Gy or 60 Gy PORT, and high-risk patients received 66 Gy PORT with concurrent weekly cisplatin. With a median follow-up of over 35 months, the trial showed excellent PFS and OS across all groups. Low-risk patients achieved a 2-year PFS rate of 97% and a 2-year OS rate of 100%, confirming outstanding outcomes even without adjuvant therapy. Among intermediate-risk patients, those receiving 50 Gy PORT (arm B) and 60 Gy PORT (arm C) had similar outcomes, with 2-year PFS rates of 95% and 96% and 3-year PFS rates of 93% and 94%, respectively. This supported the feasibility of dose reduction to 50 Gy in this group. High-risk patients treated with standard 66 Gy PORT and weekly cisplatin (arm D) achieved strong survival outcomes, with a 2-year PFS rate of 91% and a 2-year OS rate of 96%, although they experienced significantly higher toxicity rates compared to the intermediate-risk groups. The QoL outcomes showed a decline during treatment across all arms, particularly in high-risk patients (arm D). However, the QoL scores and swallowing function recovered to baseline values in arms A–C by 6 months post-treatment. Notably, 56% of patients in arms B and C had stable or improved QoL scores at 6 months, compared to 38% in arm D (*p* = 0.009). The trial confirmed that primary transoral surgery (TOS) combined with tailored PORT, including reduced-dose regimens for intermediate-risk patients, provides excellent oncologic control, improved quality of life outcomes, and favorable functional results for HPV-associated OPSCC. These findings validate a de-intensification approach for appropriately selected patients while emphasizing the importance of risk-based treatment personalization to optimize outcomes and reduce toxicity.

The AVOID [39] trial (n = 60; 2014–2017) evaluated a de-intensified PORT approach for HPV-associated OPSCC patients with resected stage pT1-pT2 N1-3 tumors (Table 1). Eligible patients had completely resected, nonmetastatic p16+ tumors without adverse features on primary tumor pathology (no perineural invasion, no lymphovascular invasion, and ≥2 mm margins) that were associated with increased local recurrence risk but with adverse features in the nodal bed, which would warrant adjuvant radiation. The patients underwent TORS and selective neck dissection, with radiation therapy (RT) limited to cervical lymph node regions while avoiding the primary tumor bed. Radiation doses were stratified based on nodal involvement, with doses of 60 Gy for involved nodes, 54 Gy for adjacent regions, and 63–66 Gy for ENE areas. With a median follow-up of 2.4 years, the trial reported a 2-year local control rate of 98%, with only one primary site recurrence, one regional recurrence (2%), and two cases of distant metastases (3%). Local recurrence-free survival (RFS) at 2 years was 98%, and OS was 100%. Late toxicities were minimal, with no persistent feeding tube dependence and only two cases (3%) of soft tissue necrosis, which resolved. The findings suggest that de-intensified PORT targeting only the at-risk cervical lymph nodes while avoiding the primary tumor bed is safe and effective for selected patients with HPV-associated OPSCC. This approach showed promise for reducing the treatment burden and toxicity and led to further investigations.

The ongoing PATHOS phase II/III trial (since 2015) is investigating the effects of risk-based, reduced-intensity adjuvant treatment in patients with HPV-associated OPSCC, confirmed by central testing of diagnostic biopsy specimens via p16 IHC and high-risk HPV in situ hybridization, at stages T1-T3 N0-N2b M0, following transoral surgery (Table 1) [40]. Post-surgical pathological assessments were used to categorize patients into low-, intermediate-, and high-risk groups, each receiving a tailored adjuvant treatment: (1) Low-risk patients without adverse histological features did not undergo further treatment; (2) intermediate-risk patients included those with T3 tumors or T1–T2 tumors with additional risk factors or metastasis in a single ipsilateral node with a diameter of 31–60 mm, or nodal metastasis in multiple ipsilateral nodes with diameters of less than 61 mm. Additionally, patients with tumors showing evidence of perineural and/or vascular invasion or with close margins (1–5 mm) around the primary tumor specimen—despite negative marginal biopsies and no evidence of cervical lymph node extracapsular spread (ECS)—were randomized to receive either standard (60 Gy) or reduced-dose radiotherapy (50 Gy) following surgery. (3) High-risk patients, defined as those with tumors of any T or N stage exhibiting specific high-risk pathological features—such as positive margins (<1 mm) around the primary tumor (with negative marginal biopsies) and/or evidence of cervical lymph node ECS—were allocated to receive either postoperative CRT with 60 Gy in 30 fractions over 6 weeks combined with concurrent cisplatin or PORT alone at 60 Gy in 30 fractions over 6 weeks. This trial is ongoing, and results are waited to inform future treatment strategies.

The MC1273 trial (NCT01932697; n = 79; 2013–2016) was a single-arm phase II trial that explored the potential of aggressive dose de-escalation in adjuvant radiotherapy (RT) for patients with IHC-confirmed p16 positive OPSCC, aiming to reduce treatment-related toxicity without compromising disease control (Table 1) [41,42]. The inclusion criteria also required patients to have completely resected tumors with clear surgical margins (R0). The patients included in the study were further stratified based on nodal involvement (N0 or N1 staging). Arm A (intermediate risk) received 30 Gy, twice daily. Arm B included patients with ENE who received the same treatment plus a simultaneous integrated boost to nodal levels with ENE to 36 Gy, twice daily. After completion of this clinical trial, researchers then compared the de-escalated adjuvant radiation therapy (DART) regimen of 30–36 Gy twice daily with concurrent docetaxel to a cohort of patients treated with standard 60 Gy once daily with or without cisplatin in post-surgical patients. In arm A, results demonstrated similar 3-year PFS rates between the de-escalation and standard therapy cohorts (87% and 90%, respectively), with no significant differences in local, regional, or distant metastasis-free survival rates. The findings for intermediate-risk HPV-associated OPSCC patients indicated that dose de-escalation may be feasible while maintaining oncologic outcomes, potentially reducing treatment-related side effects, although this does entail concurrent chemotherapy and twice daily radiation. It is notable that for patients in arm B, with high-risk factors such as pN2 disease and ENE, elevated rates of distant metastasis were found compared to the low-risk group, suggesting that treatment for these patients should not be de-escalated.

Given the need to confirm the findings of the MC1273 trial, the MC1675 De-Escalated Adjuvant Radiation Therapy (DART-HPV) phase III trial (n = 194; 2016–2020) aimed to do so. This trial compared the same 30–36 Gy DART with docetaxel treatment regimen with standard-of-care adjuvant RT. They found a significant reduction in the use of a feeding tube between the DART (2%) and standard-of-care groups (27%; *p* < 0.0001). Swallowing function was also improved in the DART group (*p* = 0.015) [43].

Collectively, the DIREKHT, ECOG-ACRIN E3311, AVOID, and DART-HPV trials demonstrated the potential of risk-adapted and de-intensified postoperative therapies to maintain excellent oncologic control while reducing treatment-associated toxicities in patients with HPV-associated OPSCC. These studies highlight the importance of stratifying patients based on tumor and nodal pathologies, surgical margins, and ENE to guide treatment decisions. By tailoring radiation doses and selectively sparing non-critical areas, these trials achieved favorable survival outcomes and improved the QoL for patients. Together, they emphasize the need for continued research to refine patient selection criteria and optimize therapeutic approaches for this unique patient population.

**Table 1:** Table of OPSCC de-intensification clinical trials.

Trial	Patient Population	Stratification Method	Intervention	Conclusions
DIREKHT (2014–2021) [37] Germany and Austria	150 HNSCC (94 OPSCCs; 77 HPV associated) Inclusion criteria: primary tumor and neck dissection with resection margin ≥1 mm; no distant metastases	Tumor stage, resection margins, and lymph node criteria	Arm A: dose reduction in the primary tumor region to 56 Gy Arm B: omission of contralateral neck radiotherapy Arm C: combination of dose reduction and omission of contralateral neck radiotherapy	De-intensified therapy maintained excellent oncologic outcomes (low locoregional recurrence (1%) in OPSCC) while reducing toxicity. Higher recurrence in oral cavity cancer highlights the need for careful patient selection.
ECOG-ACRIN E3311 (2013–2017) [38] USA	359 evaluable HPV-associated OPSCCs Inclusion criteria: T1-T2; N1-N2b	Surgical pathology (e.g., margins and ENE)	Arm A: low risk, observation Arm B: intermediate risk, 50 Gy PORT Arm C: intermediate risk, 60 Gy PORT Arm D: high risk, 66 Gy PORT + cisplatin	De-intensification (50 Gy) feasible in intermediate-risk patients, with excellent survival (2-year PFS > 94%). Observation sufficient for low-risk patients. High-risk patients need intensified treatment.
AVOID (2014–2017) [39] USA	60 HPV-associated OPSCCs Inclusion criteria: pT1-pT2 N1-3 with no adverse pathological features (e.g., PNI, lymphovascular invasion, and close margins)	Tumor and nodal pathologies	Radiation targeted cervical lymph nodes only (doses varied by involvement: 60 Gy to involved nodes; 54 Gy to adjacent regions)	De-intensified PORT to lymph nodes is safe, achieving high local control (98%) and reducing treatment burden. Toxicity was minimal.
PATHOS Trial (2015–) [40] United Kingdom	HPV-associated OPSCC Inclusion criteria: T1-T3; N0-N2b	Tumor and nodal pathologies and smoking history	Arm A: low risk, observation Arm B: intermediate risk, 60 Gy vs. 50 Gy PORT Arm C: high risk: 60 Gy PORT + cisplatin vs. RT alone	Ongoing trial aiming to assess risk-based treatment to minimize toxicity while maintaining survival.
MC1273 Trial (2013–2016) [43] USA	194 HPV-associated OPSCCs Inclusion criteria: smoking history of 10 pack years or less and negative margins	ENE for (arm A1 vs. arm A2)	Arm A1: de-escalated RT (30 Gy) + concurrent docetaxel Arm A2: de-escalated RT (36 Gy) + concurrent docetaxel Arm B: standard RT (60 Gy ± cisplatin)	De-escalation achieved similar 3-year PFS (87% vs. 90%) and comparable local/regional/distant metastasis-free survival rates. Elevated distant metastasis rates in patients with high-risk features (e.g., pN2 and ENE) indicate that treatment should not be de-escalated for these cases.
Sampieri et al., 2024 [44] (2012–2022) South Korea and Italy	300 HPV-associated OPSCCs Inclusion criteria: locoregionally advanced biopsy proven HPV; stage III-IV (AJCC-v7); successful completion of TORS	Neoadjuvant chemotherapy (NAC) use	Arm A: NAC (cisplatin and TS-1) + TORS Arm B: upfront TORS	NAC reduced pathological staging and high-risk features. NAC + TORS resulted in functional benefits by reducing need for CRT, reserved for recurrence.
Sadeghi et al., 2020 [45] (2008–2018) North America	197 HPV-associated OPCs Inclusion criteria: stage III-IV (AJCC-v7); treatment naïve; undergoing curative intent treatment	NAC use	Arm A: NAC (cisplatin and docetaxel) + TORS Arm B: concurrent chemoradiotherapy (CCRT)	NAC + TORS achieved superior 5-year DFS (96% vs. 68%) and no distant-only metastasis. Reduced toxicity compared to CCRT.
NRG Oncology RTOG 1016 (2011–2014) [46] USA and Canada	849 HPV-associated OPSCCs Inclusion criteria: stage T1–T2, N2a–N3 M0 or T3–T4, N0–N3 M0 (AJCC-v7); Zubrod performance status 0 or 1	None	Arm A: radiotherapy + cetuximab Arm B: radiotherapy + cisplatin	Cisplatin significantly outperformed cetuximab in OS, PFS, and locoregional control. Cisplatin remains the standard of care. The trial was stopped early.
TROG12.01 (2013–2018) [47] Australia and New Zealand	189 HPV-associated OPSCCs Inclusion criteria: AJCC-v7 stage III (excluding T1-2N1) or stage IV (excluding T4 and/or N3 and/or N2b-c if smoking history >10 pack years and/or distant metastases)	None	Arm A: IMRT (70 Gy/35 fractions) + cisplatin Arm B: IMRT (70 Gy/35 fractions) + cetuximab	Cisplatin superior to cetuximab in FFS and distant failure rates. No symptom burden reduction with cetuximab. Cisplatin remains preferred for low-risk cases.
NRG-HN005 (2019–2023) [48] USA	382 HPV-associated OPSCCs Inclusion criteria: stage T1-2N1M0 or T3N0-N1M0 (AJCC-v8) and ≤10 pack year smoking history	None	Arm A (control): 70 Gy IMRT + cisplatin Arm B: 60 Gy IMRT + cisplatin Arm C: 60 Gy IMRT + nivolumab	The experimental arms failed to demonstrate non-inferiority compared to arm A, which had 98% PFS rate through 2 years. The trial will not continue to phase III.
Samuels et al., 2016 (2003–2011) [49] USA	53 HPV-associated OPCs Inclusion criteria: stage III or IV, non-T4, non-N3, with little or no smoking history (≤10 pack years)	Chemotherapy use	Arm A: IMRT + carboplatin/paclitaxel Arm B: IMRT + cetuximab	No significant improvement in QOL or dysphagia with cetuximab compared to carboplatin/paclitaxel. Cetuximab not a viable de-intensification strategy for HPV-associated OPSCC.
Rosenberg et al., 2022 (2020–2022) [50] USA	46 HPV-associated OPSCCs Inclusion criteria: AJCC-v8 N1 (≥3 cm), N2-N3 nodal disease, or T3-T4 primary tumor	cfHPV-DNA reductions	Arm A: a ≥50% reduction in cfHPV-DNA levels: single-modality de-escalated treatment, such as TORS or radiation alone at 50 Gy Arm B: 30–50% underwent intermediate de-escalation (chemoradiation at 50 Gy with cisplatin) Arm C: reductions < 30% required regular-dose chemoradiation at 70 Gy	Post-treatment detectable cfHPV-DNA strongly predicted recurrence, with a sensitivity of 100% and a positive predictive value of 80% for identifying recurrences. This study is an initial demonstration of the utility of cfHPV-DNA to help stratify patients.
Lee et al., 2024 (2017–2024) [51] USA	152 HPV-associated OPSCCs Inclusion criteria: T0-2/N1-N2c	Hypoxic status determined by FMISO-PET	Arm A: nonhypoxic; 30 Gy over 3 weeks Arm B: hypoxic; 70 Gy over 7 weeks	There was a 94% PFS rate in arm A and 96% in arm B, suggesting that stratifying for hypoxic status maintains PFS.

### 2.2. Neoadjuvant Clinical Trials

Neoadjuvant clinical trials provide valuable insights into the potential of preoperative therapies to optimize outcomes for patients with locoregionally advanced HPV-associated OPSCC. A retrospective bi-centric trial (2012–2022) evaluated a neoadjuvant chemotherapy (NAC) regimen consisting of cisplatin and TS-1, which is an oral medication based on 5-fluorouracil (5-FU), combined with TORS versus upfront TORS alone for treating locoregionally advanced OPSCC (Table 1) [44]. The study included 300 patients with stage III-IV OPSCC, according to AJCC-v7, from two centers in South Korea and Italy. The study population consisted of 198 patients who received NAC followed by TORS and 102 who underwent upfront TORS alone. This trial found that p16+ OPSCC patients who underwent NAC followed by TORS had reduced pathological staging and fewer high-risk features compared to those receiving upfront surgery, with comparable survival outcomes between the two groups. Notably, 51% of the p16+ NAC group could complete their treatment with surgery alone, compared to only 16% in the upfront surgery cohort, highlighting NAC with TORS as a promising de-intensification strategy. This approach provides functional benefits by potentially reducing the need for CRT, which can be reserved for salvage treatment in case of recurrence, supporting long-term survival.

In the prospective cohort study (2008–2018) of nonmetastatic HPV-associated OPSCC patients, NAC, consisting of cisplatin and docetaxel, followed by TORS was evaluated as a treatment for locoregionally advanced cases (Table 1) [45]. The study compared 55 patients treated with NAC and surgery to a historical cohort of 142 patients who received concurrent chemoradiotherapy (CCRT). The results showed that NAC followed by TORS led to a 5-year DFS rate of 96%, which is significantly better than the 68% observed in the CCRT group. The NAC + TORS group had no cases of distant-only metastatic recurrence, whereas the CCRT group showed an 11% incidence. Additionally, none of the NAC-treated patients were feeding tube-dependent at 12 months, compared to 25% in the CCRT group [45]. These findings suggest that NAC followed by TORS is a less toxic alternative to CCRT for locoregionally advanced HPV-associated OPSCC, warranting further investigation in future trials.

### 2.3. Definitive Chemoradiation Trials

Definitive chemoradiation trials focus on the concurrent use of chemotherapy, primarily cisplatin or cetuximab, with radiotherapy to target malignant cells at and around the primary tumor site. This approach aims to enhance locoregional control, address micrometastatic disease, and improve OS, particularly in cases of locally advanced disease. Chemotherapy in this context is administered to sensitize tumor cells to radiation, maximizing the therapeutic effect and improving outcomes.

A clinical trial (NRG Oncology RTOG 1016, 2011–2014) evaluated the substitution of cisplatin with cetuximab in radiotherapy regimens for HPV-associated OPSCC patients to assess whether cetuximab could provide comparable survival benefits with reduced toxicity (Table 1). The patients were stratified into low-, intermediate-, and high-risk groups based on HPV status, smoking history, and nodal stage, with the treatment tailored accordingly. The study found that cisplatin significantly outperformed cetuximab in OS, PFS, and locoregional control, particularly in high-risk patients. The five-year OS was 85% for cisplatin versus 78% for cetuximab (HR: 1.45, 95% CI: 1.03–2.05), and cetuximab was associated with a higher risk of locoregional failure (17% vs. 10%) and worse PFS (HR: 1.72, 95% CI: 1.29–2.29). While both treatments had similar moderate-to-severe toxicity rates, cisplatin was linked to greater hematologic toxicities (e.g., anemia, nephrotoxicity, and hearing impairment), whereas cetuximab caused more acneiform rashes. Given the clear findings, cisplatin plus radiotherapy remains the standard of care for eligible patients, as it provides superior survival and disease control outcomes [46].

The TROG12.01 study (2013–2018) was a phase III randomized trial designed to compare the effectiveness and toxicity of RT combined with either weekly cisplatin or cetuximab in patients with low-risk HPV-associated OPSCC (Table 1) [47]. Ang’s low-risk criteria, identifying patients with HPV-associated tumors, lower nodal stages (below N2b), and a minimal smoking history, were applied with an additional exclusion of T4 and/or N3 cases to select low-risk, HPV-associated OPSCC patients [52]. A total of 182 low-risk, HPV-associated OPSCC participants were enrolled and randomly assigned to two treatment arms. In the first arm, the patients received IMRT at a total dose of 70 Gy, divided into 35 fractions over a 7-week period, along with weekly doses of cisplatin at 40 mg/m^2^ for a total of 7 doses. In the second arm, the patients also underwent IMRT with the same 70 Gy in 35 fractions over 7 weeks but received cetuximab instead of cisplatin. Cetuximab was administered as a 400 mg/m^2^ loading dose one week before radiotherapy, followed by weekly doses of 250 mg/m^2^ for 7 weeks. The results indicated that, again, cisplatin was more effective than cetuximab in terms of failure-free survival, with three-year failure-free survival rates of 93% for cisplatin compared to 80% for cetuximab (HR = 3.0; 95% CI: 1.2, 7.7). Additionally, freedom from distant failure at three years favored cisplatin (97% vs. 88% for cetuximab; *p* = 0.018). Although cetuximab showed slightly lower rates of some acute side effects, the overall symptom burden and late toxicity rates were similar across both treatment arms. These findings affirmed that cisplatin, due to its superior efficacy in disease control without increasing long-term symptoms, remains the recommended chemotherapeutic agent for patients with low-risk, HPV-associated OPSCC when combined with radiation therapy.

A recent clinical trial, NRG-HN005 (2019–2023), builds on prior investigations into optimizing treatment regimens (Table 1) [48]. While NRG Oncology RTOG 1016 demonstrated the superiority of cisplatin over cetuximab in concurrent radiotherapy regimens, NRG-HN005 sought to evaluate whether a reduced radiation dose or the inclusion of immunotherapy could offer comparable outcomes with potentially lower toxicity. This phase II/III non-inferiority study included patients with p16-positive OPSCC and minimal smoking history (≤10 pack years), randomizing them into three treatment arms stratified by Zubrod performance status: standard radiation (70 Gy over 6 weeks) with cisplatin, as informed by RTOG 1016, reduced-dose radiation (60 Gy over 6 weeks) with cisplatin, and reduced-dose radiation (60 Gy over 5 weeks) with nivolumab. At a median follow-up of 2.2 years, the 2-year PFS rates were 98% for the standard arm, 89% for the reduced-dose cisplatin arm, and 90% for the nivolumab arm. The study showed that the standard regimen of 70 Gy with cisplatin remains the most effective treatment, offering superior disease control compared to the alternative approaches. As a result, the trial did not advance to phase III testing. These findings reinforce the established role of cisplatin-based regimens as the standard of care for eligible patients, aligning with prior evidence from RTOG 1016, which demonstrated the consistent efficacy of cisplatin in achieving better overall survival, progression-free survival, and locoregional control. However, this trial also highlights the need for caution in treatment de-escalation strategies, given the significant PFS difference in the standard radiation dose versus the de-escalated radiation dose arms.

In a non-randomized phase II clinical trial (2003–2011), 53 patients with HPV-associated OPSCC (stage III or IV, excluding T4 and N3) and minimal or no smoking history (≤10 pack years) were assessed [49]. In the chemo-radiotherapy arm, patients received IMRT at 70 Gy over 35 fractions, combined with weekly doses of carboplatin and paclitaxel (30 mg/m^2^). In the cetuximab–radiotherapy arm, patients also received 70 Gy IMRT over 35 fractions, paired with an initial cetuximab loading dose of 400 mg/m^2^ followed by weekly doses of 250 mg/m^2^. By comparing dysphagia, QoL, and other toxicity outcomes, the study explored whether cetuximab can offer effective treatment with potentially fewer side effects than traditional chemotherapy. The results showed that both groups experienced similar degrees of dysphagia and QoL impairment immediately post-treatment, which partially improved by 12 months. No significant differences were found between the two treatment groups across QoL domains, including eating, xerostomia, and swallowing function, suggesting comparable impacts on patient-reported outcomes and clinical measures of dysphagia.

While cetuximab was initially proposed as a less toxic alternative to cisplatin, clinical trials have consistently shown that it is associated with worse survival outcomes and no meaningful improvement in toxicity or QoL. These results confirm the importance of maintaining cisplatin-based CRT as the gold standard for eligible patients and highlight the need for further investigation into alternative strategies to reduce toxicity while preserving efficacy in HPV-associated OPSCC.

### 2.4. Biomarker-Driven Trials

Plasma cell-free HPV-DNA (cfHPV-DNA) levels have demonstrated a strong correlation with treatment response in HPV-associated OPSCC, offering a measurable biomarker for guiding adaptive treatment strategies. In a prospective phase I study involving 46 patients with locoregional HPV-associated OPSCC treated between 2020 and 2022 (NCT04572100), those achieving a ≥50% reduction in cfHPV-DNA levels after three cycles of neoadjuvant chemotherapy were eligible for single-modality de-escalated treatment, such as TORS or radiation alone at 50 Gy, achieving a 1-year PFS of 100% (Table 1). Patients with cfHPV-DNA reductions of 30–50% underwent intermediate de-escalation (chemoradiation at 50 Gy with cisplatin) and also achieved a 1-year PFS of 100%, while those with reductions below 30% required regular-dose chemoradiation at 70 Gy, with a lower 1-year PFS of 75%. The clearance of cfHPV-DNA after two cycles of chemotherapy was associated with a 1-year PFS of 100%, compared to 79% for patients with detectable cfHPV-DNA (*p* = 0.1). Post-treatment detectable cfHPV-DNA strongly predicted recurrence, with a sensitivity of 100% and a positive predictive value of 80% for identifying recurrences. These findings highlight cfHPV-DNA as a promising, non-invasive biomarker for response-adaptive therapy, enabling tailored treatment intensity to optimize survival while minimizing treatment-related toxicity [50]. Further validation with extended follow-up is anticipated.

In a phase II trial, F-fluoromisonidazole positron emission tomography (FMISO-PET) was used to stratify patients with tumors containing less hypoxia who could be treated with significantly lower doses of radiation in conjunction with high-dose cisplatin (NCT03323463) [51]. Of 152 patients with T0-2, N1-2 disease who were enrolled, 128 had nonhypoxic tumors and, thus, met the criteria for significant de-escalation, with treatment consisting of 30 Gy over 3 weeks (Table 1). The remaining patients who had hypoxic tumors were treated with a standard regimen of 70 Gy over 7 weeks. There was a 94% 2-year PFS rate in the 30 Gy cohort and 100% 2-year OS, pointing to a promising area of treatment de-escalation in patients with nonhypoxic tumors.

### 2.5. Challenges and Future Directions

De-intensification trials in HPV-associated OPSCC aim to balance oncologic control with reduced treatment-related toxicity. These efforts reflect a broader push toward precision medicine, incorporating risk stratification and adaptive therapeutic strategies. While promising results have emerged, several challenges and opportunities remain for refining these approaches to avoid any increase in mortality while further improving QoL for survivors.

A primary challenge lies in the accurate stratification of patients. Current methods rely on clinical factors such as the tumor stage, nodal involvement, and smoking history. Although these criteria have demonstrated utility, limitations in predicting individual responses remain. For example, while trials like ECOG-ACRIN E3311 stratified patients effectively into low-, intermediate-, and high-risk groups, a subset of intermediate-risk patients may still face overtreatment or undertreatment due to variability in tumor biology [38]. The integration of biomarkers such as plasma cfHPV-DNA offers potential but remains underutilized. While cfHPV-DNA levels have shown correlations with treatment response and recurrence risk in OPSCC, their routine clinical application is hindered by the lack of standardization in measurement techniques and thresholds [50].

## 3. Candidate Molecular and Cellular Biomarkers for Risk Stratification

The integration of advanced molecular profiling is a pivotal area for improvement in risk stratification. Multi-omics approaches, combining genomic, transcriptomic, and epigenomic data, could enhance patient stratification and uncover novel therapeutic targets. Such techniques may help identify specific subgroups within HPV-associated OPSCC that could benefit most from de-intensified therapies [53,54]. This strategy could potentially further reduce overtreatment and identify patients at risk of recurrence earlier. Unlike traditional staging and clinical factors, molecular markers can capture unique aspects of tumor biology, immune response, and viral integration patterns specific to HPV-driven cancers. By integrating biomarkers, such as DNA methylation signatures, gene expression profiles, and immune cell infiltration metrics, clinicians can move beyond conventional staging to predict treatment response and long-term outcomes with greater precision. This section explores current advancements in molecular biomarkers for HPV-associated OPSCC, their clinical implications, and future directions for translating these insights into routine patient care.

### 3.1. E6*/FL Ratio

The expression of the main oncogenes of HPV, E6 and E7, is required for tumorigenesis, but there is evidence that E6 isoform variations have varying effects on tumor progression. E6 has a full-length variant (E6FL) expressed in all HPV types, but in high-risk HPV types, E6 is also expressed in shorter spliced isoforms collectively known as E6*. The most common variant of E6* is E6*I with the removal of 183bp of E6FL in HPV-16. An early study by Pang et al. found that OPSCC cell lines that express E6*I were more sensitive to radiation compared to controls [55]. In another study by Sannigrahi et al., only E6FL was associated with a reduction in oxidative phosphorylation, which was then shown to be associated with improved OS in multiple cohorts of HPV-associated OPSCC, including in TCGA (*p* = 0.019) [55,56]. Looking further into the downstream effects of E6* in patient samples, Qin et al. developed an E6*:E6FL influence score that uses genes significantly correlated with the ratio of E6* to E6FL (Table 2). This influence score was significantly negatively associated with oxidative phosphorylation pathways (FDR = 1.86 × 10^−26^) [57]. Qin et al. identified that a high E6*:E6FL influence score resulted in worse survival even when correcting for sex, age, tumor stage, tumor site, and smoking status (n = 54 HPV-16+; Cox hazard regression, *p* = 0.02; HR = 0.2). Overall, these studies suggest that a higher ratio of E6* to E6FL is worse for the survival of patients potentially due to the increased effects of E6* on oxidative phosphorylation and/or its influence on E7 protein expression. E6* has been shown to enhance E7 translation by extending the mRNA region between the E6 stop codon and the E7 start codon, facilitating ribosome assembly [58]. Increased E7 levels have been linked to recurrence in HPV-associated OPSCC. In a study with 52 HPV-associated OPSCCs, patients with recurrence exhibited significantly higher serum antibodies to E7 (*p* = 0.002) and reduced E7 antibody clearance compared to disease-free patients (*p* = 0.0016) [59]. However, the relationship between E6* splicing and E7 expression remains debated, as other studies suggest no direct effect of E6* splicing rates on E7 expression [60].

**Table 2:** Table of biomarkers that have shown significance with outcomes in HPV-associated OPSCC.

Study	Biomarker	Patient Cohort	Measure of HPV Used in Study	Biomarker Type	Results
Qin et al., 2020 [57]	E6*:E6FL influence score	54 HNSCC	RNA-seq	RNA-seq gene set score	High E6*:E6FL influence score had worse OS (*p* = 0.02)
Spector et al., 2016 [59]	E7 serum antibodies	52 OPSCC	PCR	Enzyme-linked immunosorbent assay (ELISA) from blood	Recurrent patients were more likely to have E7 serum antibodies (*p* = 0.002)
Koneva et al., 2018 [61]	HPV integration	66 HNSCC	RNA-seq	RNA-seq	Non-integrated (episomal) had better OS (*p* = 0.0436)
Nulton TJ, et al., 2018 [62]	E2/E7	56 HNC	RNA-seq	RNA-seq gene expression score	Lower E2/E7 ratio had worse survival (30% 5 yr. OS) compared to higher E2/E7 (72% 5 yr. OS), *p* = 0.034
Misawa K, et al., 2020 [63]	CALML5, DNAJC5G, and LYD6D methylation	35 OPC	PCR and p16 IHC	DNA methylation	DFS was significantly shorter when GPT, LY6D, MAL, and MRGPRF were methylated (*p* = 0.021, *p* = 0.019, *p* = 0.012, and *p* = 0.007, respectively)
Chen J, et al., 2024 [64]	SNV1339A>G, SNV1950A>C, and SNV4298A>G	40 OPC cases	HPV16 WGS	SNV	High-risk HPV SNVs were associated with substantially poorer 3-year OS; patients with at least one high-risk SNV had a median survival of 3.6 years, compared to 4.2 years for those without any of these variants (HR = 1.29 × 10^3^; *p* < 1.0 × 10^−6^)
Ward et al., 2013 [65]	Tumor-infiltrating lymphocytes	149 OPSCC	HPV ISH	H&E imaging	High TIL was better for DSS (*p* < 0.001); TIL levels predicted similarly for PFS (*p* < 0.001)
Nordfors et al., 2013 [66]	CD8+ cells	216 OPSCC	DNA	Immunohistochemistry	High CD8+ was better for OS (*p* < 0.001) and DFS (*p* = 0.004)
Van Abel et al., 2020 [67]	T-cell fraction	65 OPSCC	HPV ISH	Immunosequencing	High TCF was a strong predictor of PFS (*p* = 0.02) and CSS (*p* < 0.05)
Hong et al., 2019 [68]	PDL1	81 OPSCC	DNA and p16 IHC	Immunohistochemistry	PDL1 negative cases showed more locoregional recurrence (*p* = 0.027) and worse OS (*p* = 0.008)
Solomon et al., 2018 [69]	PDL1	190 OPSCC	p16 IHC	Immunohistochemistry	High PDL1 expression was associated with OS (*p* = 0.023)
Solomon et al., 2018 [69]	CD8+ cells	190 OPSCC	p16 IHC	Immunohistochemistry	High CD8+ TIL abundance was associated with improved OS (*p* = 0.017)
Zeng et al., 2022 [70]	UWO3	906 OPSCC	p16 IHC, PCR, and DNA	RNA-seq gene set score	High UWO3 had better DFS than low and moderate UWO3 (*p* = 3.6 × 10^−5^; *p* = 0.006)
Locati et al., 2019 [71]	CI1/CI2/CI3 subtype	346 HNSCC	DNA (59.5%); RNA-seq (28%); Other (12.5%)	Microarray subtyping	CI1 had better OS than CI3 or CI2 (*p* = 4.76 × 10^−9^)

### 3.2. HPV Expression

Several studies have assessed whether the expression of certain HPV genes is linked to better survival and treatment response in HPV-associated HNSCC, specifically using the ratio of E2, E6, or E7 gene expression, copy number, and viral integration status to predict patient outcomes [52,72,73]. The HPV genome can exist in an episomal, non-integrated state or an integrated state, where the virus incorporates all or part of its own material into the host’s genome [74]. In episomal HPV, late-stage infections within differentiating keratinocytes experience activation of the late promoter of E7, leading to high levels of E1, E2, and late gene product expression [75]. However, integration often disrupts the E2 gene, which is responsible for regulating E6 and E7. When E2 is disrupted, E6 and E7 expressions increase, leading to greater oncogenic activity and genetic instability [76]. The scenario is complicated by the fact that a patient can experience a mixed state where episomal and integrated DNA exist concurrently [77]. Thus, the ratio between E2 and E6 (or E7) expression in the context of integration status has been tested as a biomarker in HPV-related cancers. In cervical cancer, the ratio of HPV-16 E2 to E6 has been established as a prognostic biomarker, as studies demonstrate that a decrease in E2/E6 predicts cervical lesion progression and, thus, a poorer prognosis [78,79].

In HNSCC, the HPV integration status has been directly used in stratifying HPV-associated HNSCC [61]. Using TCGA survival data, integration-negative patients demonstrated markedly better OS than integration-positive patients, with log-rank p-values showing significant differences in two groups (*p* = 0.044) and three groups with HPV-negative (*p* = 0.0158) comparisons [61]. Notably, the survival rates for integration-positive tumors were comparable to HPV-negative tumors, suggesting that HPV genome integration undermines the survival advantage typically associated with HPV positivity. Cox regression analysis confirmed that integration status was a significant independent predictor of OS, even after adjusting for clinical covariates such as age, smoking status, and cancer stage [61].

However, an earlier study involving 179 HPV-associated OPSCC patients showed that the E2/E6 ratio did not have significant results specifically for OPSCC (Table 2). In this study, outcomes were compared based on the physical state of HPV, which was determined using the E2/E6 copy number ratio as a biomarker to classify HPV as episomal, mixed (episomal and integrated), or integrated [80]. For the episomal HPV group (n = 22), the 3-year survival rates were 91% for OS, 95% for DSS, and 80% for PFS. The mixed HPV group (n = 115) had 3-year survival rates of 95% for OS, 97% for DSS, and 93% for PFS. Finally, the integrated HPV group (n = 42) had 3-year survival rates of 88% for OS, 90% for DSS, and 85% for PFS. No significant differences were observed in OS (*p* = 0.101), DSS (*p* = 0.105), PFS (*p* = 0.23), or distant metastasis (*p* = 0.76) between the groups. Although patients with mixed HPV had a trend toward better survival outcomes, these differences were not statistically significant.

In a study involving 56 HPV16-positive HNC patients and 340 HPV-negative HNC patients, the ratio of E2 to E7 expression was used as a biomarker to infer the integration status of the HPV genome [62]. Patients with an E2/E7 ratio < 0.02 were classified as having integrated HPV, whereas those with a higher E2/E7 ratio (mean = 0.15) were identified as having episomal HPV. Patients classified as episomal had a 72% 5-year survival rate, which was significantly better than the 30% survival rate for patients with integrated HPV (*p* = 0.034). Furthermore, there was no significant survival difference between integrated HPV patients and non-HPV patients (*p* = 0.265), whose 5-year survival rate was 40%. This suggests that intact E2 expression may confer a less aggressive tumor phenotype, whereas the loss of E2 could lead to uncontrolled oncogene expression and worse outcomes. It is important to note that loss of E2 expression or decrease in the E2/E7 ratio is associated to but not synonymous with integration.

A study involving 31 OPSCC and 17 non-OPSCC HNSCC samples found that HPV-associated OPSCC patients with an intact E2 gene not only had a higher viral load but also demonstrated regulated expression of the E6 and E7 oncogenes, which correlated with improved outcomes [81]. Specifically, when compared to tumor samples with disrupted E2, OPSCC possessing an intact E2 had a lower risk of locoregional recurrence (log-rank *p* = 0.04) and improved DSS (*p* = 0.03), which again suggests that the intact form of E2 may serve as a protective factor, possibly due to its ongoing regulatory role over E6 and E7. However, regardless of E2 status, high E7 and E6 expressions were associated with a lower risk of locoregional recurrence (*p* = 0.004 and 0.006, respectively), indicating that while E2 disruption is important, the levels of E6 and E7 may play an independent role in shaping clinical outcomes. Such research provides a basis for utilizing HPV viral gene profiles—specifically E2 integrity and the expression levels of E6 and E7—as prognostic tools for HPV-associated OPSCC.

### 3.3. DNA Methylation

Epigenetic changes in DNA methylation, including global hypomethylation and specific promoter hypermethylation, have been used as prognostic biomarkers for several types of cancers, such melanomas, lymphomas, colorectal, liver, gastric, and breast cancers [82,83]. In HNSCC, specific DNA methylation changes can distinguish tumor subsites; for example, one study demonstrated that *SFRP1* and *CALCA* methylation are characteristic of OPSCC, while *DAPK1* hypermethylation is more frequent in laryngeal cancers [84]. A recent study combined differential methylation data with clinical information to develop predictive risk score (RS) signatures based on methylation sites in overall HNSCC, such as a novel four-gene methylation signature (*ZNF10*, *TMPRSS12*, *ERGIC2*, and *RNF215*) associated with differential survival outcomes in HNSCC patients undergoing radiotherapy [85]; however, they were unable to develop separate scores based on HPV status. Thus, much remains unknown regarding the DNA methylation sites specifically affecting clinical outcomes in HPV-associated OPSCC, and examining HPV-associated OPSCC as its own unique disease in this respect is a promising avenue of research [86].

Other studies have focused on differential DNA methylation in circulating tumor DNA (ctDNA) as a biomarker to provide non-invasive and early prediction of patient prognosis while providing information on cell origin [87,88]. Specifically, genes including *CALML5*, *DNAJC5G*, and *LYD6D* show altered methylation patterns associated with DFS in OPC [63]. Eight pre-treatment ctDNA samples showed *CALML5*, *DNAJC5G*, and *LY6D* methylation in 100%, 87.5%, and 87.5% of cases, respectively, while methylation persisted in only 25%, 0%, and 12.5% of the final samples. Moreover, hypermethylation of *CALML5* (HR = 7.01, 95% CI: 1.01–48.66) and *LY6D* (HR = 10.69, 95% CI: 1.67–68.33) was significantly associated with reduced DFS in HPV-associated OPC. These methylation biomarkers demonstrated high specificity in distinguishing cancer patients from healthy individuals. Thus, methylation of ctDNA could serve as biomarkers for tumor origin or disease progression and facilitate real-time monitoring of treatment efficacy.

### 3.4. HPV Single-Nucleotide Variations

The requirement for tumor biopsy or isolation of circulating tumor cells for HPV-associated OPSCC patients can be bypassed if single-nucleotide variations (SNVs) are used as biomarkers, similar to the use of FDA-recommended SNVs in leukemia, colorectal cancer, and breast cancer [89], as well as those currently being validated for pancreatic cancer [90,91]. However, there is a dearth of research with regard to SNVs in the context of HPV-associated OPSCC, especially with regard to viral SNVs. In a recent study of 40 HPV16+ OPC cases, three high-risk HPV SNVs (SNV1339A>G, SNV1950A>C, and SNV4298A>G) were identified, each associated with substantially poorer 3-year OS (Table 2) [64]. These SNVs were significantly linked with alterations in E1-E2 interactions, and patients with at least one high-risk SNV had a median survival of 3.61 years compared to 4.20 years for those with none of these variants (HR = 1.29 × 10^3^; *p* < 1.0 × 10^−6^). The exploration of human and viral SNVs in HPV-associated OPSCC presents a unique opportunity to discover new biomarkers for predicting patient outcomes, especially given the limited research conducted in this area.

### 3.5. Immune Infiltration Scores

The immune system’s role in cancer prognosis has long been acknowledged, but its significance and clinical application were greatly enhanced with the introduction of ICIs, such as anti-PD-L1 and anti-PD-1 [92]. These therapies demonstrated superiority over standard chemotherapy regimens in several clinical trials, particularly in the treatment of recurrent and metastatic cancers [38,93]. However, immune response in patients is not uniform, and thus, identifying biomarkers for immune infiltration has emerged as a key research area in HPV-associated OPSCC. One of the earliest papers to examine the effect of immune infiltration on patient outcomes in HPV-associated OPSCC was by Ward et al. [65]. Using a cohort of 149 HPV-associated OPSCC patients, pathologists categorized tumors as having high, moderate, or low tumor-infiltrating lymphocytes (TILs). They found a highly significant difference in both HPV-associated OPSCC DSS (log-rank *p* < 0.001) and PFS (log-rank *p* < 0.001), with higher TIL levels linked to longer survival. In another 2013 study, Nordfors et al. identified immune infiltration through immunohistochemistry of CD8+ and CD4+ cells (Table 2) [66]. In a cohort of 216 HPV-associated OPSCC patients, high CD8+ was found to be prognostically favorable in both OS (*p* < 0.001) and DFS (*p* = 0.004). These associations held true when adjusted for stage and age. They found a similar trend, but no significance in CD4+ cells. In 2020, Van Abel et al. found that the T-cell fraction (TCF) was significantly associated with HPV-associated OPSCC using a cohort of 65 patients [67]. To determine TCF, they used T-cell immunosequencing and then determined the proportion of T cells relative to the total number of nucleated cells. They found that TCF was a significant predictor of both PFS (HR 0.80, *p* = 0.02) and DSS (HR 0.69, *p* < 0.05). Overall, there is strong evidence that higher TILs in HPV-associated OPSCC is associated with improved survival.

Another immune marker is Programmed Death Ligand 1 (PDL1). PDL1 is a cell surface protein known to regulate the immune response in many cancers and is expressed on both tumor cells and a variety of immune cells, while PD-1 is primarily found on immune cells, especially exhausted T cells. The PD-1 inhibitors pembrolizumab and nivolumab are widely used in patients with recurrent metastatic HNSCC, particularly in patients who have high expression of PD-L1, as evaluated using the CPS [94]. Using a cohort of 81 patients with HPV-associated OPSCC, Hong et al. examined the role of PDL1 using immunohistochemistry staining [68]. They found that PDL1 expression significantly impacted the outcome, with low-expressing PDL1 cases being more likely to develop a locoregional recurrence (HR 4.16, *p* = 0.027), have an event defined as recurrence or death of any cause (HR 2.5, *p* < 0.05), and die (HR 3.16, *p* = 0.008). In another study, Solomon et al. found that in a cohort of 190 HPV-associated OPSCC patients, high PDL1 expression was significantly associated with improved OS (HR = 0.37, *p* = 0.023) [69]. In addition, they observed that high CD8+ abundance was associated with improved OS (HR, 0.4, *p* = 0.017), validating the earlier finding of Nordfor et al. In a meta-analysis of 1522 OPSCC patients from 12 studies, Polesel et al. found that HPV-associated OPSCC patients in particular showed better OS when comparing high vs. low PDL1 expression [95]. Further emphasizing its importance, PDL1 has been shown to be a hotspot for HPV integration in HNSCC, linked to increased PDL1 expression in tumors with nearby integration events [61,96]. Taken together, these findings suggest that the expression of PDL1 plays a key role in the immune response to HPV-associated OPSCC, making it a potentially important biomarker beyond use for predicting ICI response.

Driven by the success of PDL1 and CD8+ as predictors of survival in HPV-associated OPSCC, a more recent study by Zeng et al. developed a three-gene immune score based on the expressions of CD3E, IRF4, and ZAP70, called UWO3 [70]. UWO3 was associated with DFS in six independent cohorts comprising 906 patients. Dividing the cohort of patients into three groups based on UWO3 expression, they found that the immune-rich group had better DFS compared to both the immune-desert (HR = 9.0, *p* = 3.6 × 10^−5^) and mixed groups (HR = 6.4, *p* = 0.006), even after adjusting for age, sex, smoking status, and AJCC v8 clinical stage. The genes in this score are representative of tumor biology; CD3E is part of the T-cell receptor complex, ZAP70 plays an important role in T-cell receptor signaling, and IRF4 is a regulatory transcription factor involved in the development of immune cells.

### 3.6. Whole Genome/Transcriptome Subtyping

Stratifying patients by tumor molecular subtypes based on multi-omics data has emerged as an approach for predicting outcomes and guiding treatment. HPV-associated HNSCC exhibit substantial heterogeneity in molecular characteristics, which affects disease progression and the response to therapy. Molecular subtypes of HPV-associated HNSCC demonstrate significant variations in immune characteristics, mesenchymal features, and keratinization that correspond with distinct prognoses and recurrence risks. Since 2007, a number of studies have defined subtypes within HPV-associated HNSCC using gene expression microarray analysis, finding distinctions within HPV-associated HNSCC samples that did not rely solely on HPV expression [97,98]. This early work suggested that HPV-associated tumors are heterogeneous, but due to the low-plex methods used, these early studies could not fully characterize the differences driving these tumor subtypes. The Cancer Genome Atlas (TCGA) presented RNA-seq data from 36 HPV+ and 243 HPV− HNSCC tumors [99], classifying tumors as atypical, basal, classical or mesenchymal [100]. Because this was done agnostic to HPV status, they found that most HPV-associated tumors were classified as the atypical subtype and were unable to identify subtypes within HPV-associated HNSCC. Motivated by these established HNSCC subtypes, later studies looked within HPV-associated tumors.

The first to look within HPV-associated HNSCC using whole transcriptomic data were Zhang et al. who identified two HPV-associated subtypes using a cohort of 18 new patients and 66 TCGA patient samples [100,101]. Consistent with the previous studies using microarray data, Zhang et al. identified two subtypes, which they called IMU and KRT. IMU was shown to be more immunogenic, with more mesenchymal differentiation, and more epithelial-to-mesenchymal (EMT) differentiation signatures. The KRT subtype was identified by strong keratinization and more HPV integration events into the host genome. The KRT subtype also showed an insignificant trend toward worse survival (*p* = 0.3). In 2019, Locati et al. combined microarray and RNA-seq data from 11 distinct studies (Table 2) [71]. They defined three subtypes of HPV-associated tumors: CI1 was most immune strong, CI2 showed characteristics of high keratinization, EMT, and hypoxia, and CI3 was also highly keratinized and proliferation-related. CI1 patients had significantly better survival, with a 5-year survival probability of 0.809 vs. 0.47 and 0.197 for CI3 and CI2 patients, respectively (CI1/CI2/CI3: *p* = 4.76 × 10^−9^). The Locati and Zhang studies showed overall concordance by separating HPV-associated tumors into subtypes based on immune and keratinization characteristics. These insights underscore the prognostic potential of molecular profiling in HPV-associated HNSCC, providing rationale for more targeted therapeutic strategies.

## 4. Conclusions and Future Directions

The treatment landscape for HPV-associated OPSCC is rapidly evolving, driven by the need to maintain excellent therapeutic outcomes while minimizing treatment-related toxicities. However, significant challenges remain in refining approaches to identify and appropriately treat specific patient subgroups, particularly those at low risk. Much of the research highlighted in this review was conducted on small cohorts of fewer than 100 patients, lacked diversity, and often combined data from HPV-associated and HPV-negative OPSCC or other HNSCC sites. This severely limits the field, as the differences between HPV-associated and HPV-negative OPSCC have been demonstrated repeatedly, and studies conducted agnostic to HPV status make it challenging to draw conclusions about either cohort of patients. Other studies were based on the p16 status in the absence of measuring HPV DNA or HPV expression. These methodological limitations represent persistent challenges in the field. Larger, multicenter studies with enhanced collaboration across institutions and confirming HPV-associated status as inclusionary criteria are critical to validate promising findings, improve risk stratification methods, and refine treatment protocols.

An important area of focus is the distinction between de-escalation and precision medicine. De-escalation emphasizes reducing the intensity of standard treatments to minimize adverse effects, often applied across relatively broad patient groups. Precision medicine, on the other hand, tailors treatments based on individual molecular and clinical characteristics. However, the field must be vigilant and learn from previous clinical trial failures; the de-escalation of any subgroup of HPV-associated OPSCC without accurate risk classifications for precision medicine risks the loss of additional lives. The improved integration of precision medicine approaches into de-escalation strategies will be pivotal in this patient-centered paradigm.

The accurate identification of HPV-associated tumors still remains a pressing issue, given the limitations of current diagnostic tools. While p16 IHC is a widely used surrogate marker for HPV positivity, neither its sensitivity nor specificity are 100%, with estimates of specificity being lower than for sensitivity. This is especially problematic in populations with low attributable fractions of HPV in OPSCC with subsequent low positive predictive values. This, in turn, can lead to an unacceptably high risk of under- or overtreatment of p16+/HPV− and p16−/HPV+ patients, respectively. p16−/HPV+ patients may receive harsher treatments, resulting in more adverse effects, while p16+/HPV− patients risk being under-treated and more likely to recur. These p16/HPV discordant patients also complicate the interpretation of results from clinical trials that used the p16 status without confirming HPV DNA or RNA. To address these pitfalls, there is a pressing need to consistently incorporate more precise molecular diagnostics, such as HPV DNA or RNA testing, into clinical workflows.

Future directions include the need for larger, multicenter studies to validate candidate biomarkers and risk stratification tools, especially in the context of low-attributable fraction patient populations. Promising candidates, such as plasma cfHPV-DNA dynamics, epigenetic signatures, and genomic alterations, need further investigation in diverse cohorts integrated with current state-of-the-art treatment protocols and clinical risk stratifications to ensure their clinical utility. Furthermore, promising candidate biomarkers discussed in this review—such as HPV gene-based scores like E2/E6 or E6*/FL or immune infiltration scores like CD8+, PDL1, or UWO3—need to be tested in clinical trials as methods for stratification and, in some cases, first translated into a clinical assay. These markers have shown the ability to stratify patients by outcome and, thus, can discern patients for de-intensified treatment. The continued use of clinical methods of stratification like tumor staging or nodal involvement may result in failed attempts to de-escalate not due to treatment but instead due to patient selection.

Simultaneously, there is a need to further develop low-risk-specific treatments, such as minimally invasive surgeries, reduced radiation doses, and tailored systemic therapies, to optimize the outcomes for the low-risk subgroup. Given the heterogenous epidemiology of OPSCC in the context of HPV, incorporating individual factors such as smoking, comorbid conditions, and alcohol use may allow for even finer precision in treatment. Addressing these critical gaps will advance the field toward a treatment model that is not only effective but also minimally harmful, ultimately improving the outcomes and quality of life for the growing number of patients with HPV-associated OPSCC.
